# Increased Expression of Fibulin-1 Is Associated With Hepatocellular Carcinoma Progression by Regulating the Notch Signaling Pathway

**DOI:** 10.3389/fcell.2020.00478

**Published:** 2020-06-16

**Authors:** Jiao Gong, Yusheng Jie, Cuicui Xiao, Wenying Zhou, Xinhua Li, Yaqiong Chen, Yuankai Wu, Jing Cao, Qi Zhang, Zhiliang Gao, Bo Hu, Yutian Chong

**Affiliations:** ^1^Department of Laboratory Medicine, Key Laboratory of Liver Disease of Guangdong Province, Third Affiliated Hospital of Sun Yat-sen University, Guangzhou, China; ^2^Department of Infectious Diseases, Key Laboratory of Liver Disease of Guangdong Province, Third Affiliated Hospital of Sun Yat-sen University, Guangzhou, China; ^3^Cell-gene Therapy Translational Medicine Research Center, Key Laboratory of Liver Disease of Guangdong Province, Third Affiliated Hospital of Sun Yat-sen University, Guangzhou, China

**Keywords:** Fibulin-1, hepatocellular carcinoma, apoptosis, serum marker, circulating RNA

## Abstract

Fibulin-1, a component of the extracellular matrix (ECM), its prognostic, pathophysiologic and diagnostic role in hepatocellular carcinoma (HCC) is still unexplored. We first found that either Fibulin-1 messenger RNA (mRNA) or protein level was highly elevated in HCC tissues compared with normal tissues. Fibulin-1 correlated with poor overall survival, and it was an independent prognostic predictor (*p* = 0.001). Furthermore, Overexpression or inhibition of Fibulin-1 reduced or sensitized HCC cells to apoptotic signals, and Fibulin-1 silencing suppressed the ability of HCC cells to form tumors *in vivo*. Moreover, Fibulin-1 inhibited apoptosis via the Notch pathway while Fibulin-1 silencing had no obvious effect on p-MAPK, p-c-jun and p-stat3 expression, and both Mcl-1 and Bcl-xL are targets of Fibulin-1. Furthermore, the stromal and immune score was elevated in high Fibulin-1 tissues, and FBLN1 expression was associated with increased infiltrating macrophages using xCell, TIMER and TISDIB tool based on TCGA HCC database. Importantly, the circulating cell-free RNA (cfRNA) level of Fibulin-1 in the serum were significantly increased in patients with HCC compared with those in healthy controls, individuals with chronic hepatitis B and patients with HBV-induced liver cirrhosis. The area under receiver operating characteristic curves (AUC) was 0.791 for Fibulin-1, 0.640 for α-fetoprotein and 0.868 for the combination of the two tumor markers. Our findings indicate that Fibulin-1 may be a potential prognostic indicator, a promising serum biomarker and a therapeutic target in patients with HCC.

## Introduction

Hepatocellular carcinoma (HCC), one of the most common cancers, is the main cause of cancer-related death worldwide (Ghouri et al., 2017). HCC has the following characteristics: rapid progress, resistance to anticancer therapy, high post-surgical recurrence and poor prognosis (Villanueva et al., 2013). Many advances in identifying the risk factors for HCC and understanding the pathogenesis of HCC have been made, however, the molecular mechanisms underlying hepatocarcinogenesis are still largely unknown.

The extracellular matrix (ECM) is a non-cellular structure that is present in all tissues and is essential for life, which provides physical support for tissue integrity and elasticity by regulating cell adhesion, migration, proliferation, apoptosis, survival and differentiation (Hynes, 2009; Bonnans et al., 2014). Moreover, the ECM is a vital component of the tumor microenvironment (Hynes, 2009; Buchheit et al., 2014). Basically, cancer cells need to develop resistance to anoikis, which is defined as the induction of apoptosis owing to detachment from the ECM, to promote tumor progression (Frisch and Screaton, 2001; Simpson et al., 2008). More importantly, the ECM also promotes tumor progression by increasing cell survival, providing a growth advantage and inducing angiogenesis in cancer cells (Poola et al., 2005; Cheresh and Stupack, 2008; Levental et al., 2009; Burnier et al., 2011). In addition, several studies put forward the important role of the ECM in metastasis by promoting tumor growth in the metastatic niche (Tozluoglu et al., 2013; Monboisse et al., 2014).

Fibulins are a newly recognized family of ECM proteins that modulate cell proliferation and migration (Timpl et al., 2003). Fibulin1-7, have elongated structure and many calcium-binding sites, which are based on tandem arrays of epidermal growth factor-like domains (Timpl et al., 2003). Fibulin-1 could bind to some ECM proteins, such as laminin-1 and fibronectin, and then mediate signal transduction, which regulates cell morphology, mobility and proliferation (Barth et al., 1998; Cooley et al., 2008). Furthermore, Fibulin-1 is associated with tumor development (Moll et al., 2002; Greene et al., 2003; Zhu et al., 2015). The alternative splicing of Fibulin-1 transcripts result in four separate variants termed-1A to -1D, which differ at the C-terminus of the protein. The overexpression of Fibulin-1D reduces anchorage-independent growth in fibrosarcoma-derived cells and inhibits papillomavirus-E6 protein-mediated transformation (Qing et al., 1997; Du et al., 2002). Interestingly, Fibulin-1 is increased in ovarian and breast cancers (Roger et al., 1998; Forti et al., 2002; Greene et al., 2003; Pupa et al., 2004). It was reported promoter hypermethylation of Fibulin-1 was related to tumor progression in HCV-related HCC (Kanda et al., 2011). However, to date, there is no study addressing the anti-apoptotic effect of Fibulin-1 in HBV-related HCC, and its molecular mechanisms are still essentially unclear.

In our research, Fibulin-1 was upregulated in the majority of the examined HCC tissues. Fibulin-1 silencing significantly sensitized HCC cells to apoptotic signals, and decreased the ability of HCC cells to develop tumors *in vivo*. Furthermore, Fibulin-1 inhibited apoptosis via the Notch pathway, and both Mcl-1 and Bcl-xL are targets of Fibulin-1. In addition, high Fibulin-1 expression was associated with a low rate of overall survival and Fibulin-1 was an independent prognostic predictor for overall survival. Moreover, the serum Fibulin-1 levels were significantly elevated in HCC patients than in healthy controls, chronic hepatitis B patients and HBV-induced liver cirrhosis patients. Our findings highlight the importance of Fibulin-1 dysfunction in hepatocarcinogenesis and also indicate that Fibulin-1 may be a potential prognostic indicator, a promising serum biomarker and a therapeutic target in patients with HCC.

## Materials and Methods

### Reagents

The antibodies used for western blotting were as follows: rabbit polyclonal antibodies (rAb) against Fibulin-1 (sc-20818, Santa Cruz Biotechnology, Santa Cruz, CA, United States), cleaved Notch1 (#4147, Cell Signaling Technology, Beverly, MA, United States), Mcl-1 (sc-819, Santa Cruz Biotechnology), Bcl-xL (#2764, Cell Signaling Technology), Hes1 (#11988, Cell Signaling Technology), and caspase-3 (#9662, Cell Signaling Technology) and a mouse monoclonal antibody (mAb) against β-actin (sc-81178, Santa Cruz Biotechnology). All other reagents are purchased form Sigma, unless indicated otherwise. An enhanced chemiluminescence (ECL) kit (Pierce, Rockford, IL), 4′-6’-diamidino-2-phenylindole (DAPI; Sigma-Aldrich, St. Louis, MO, United States) and cell culture plates (Corning Glass Works, Corning, NY, United States) were also used in this study.

### Tissue Specimens and Cell Lines

Nineteen paired HCC and adjacent non-tumor liver tissues were collected from patients who underwent surgical resection at the Third Affiliated Hospital of Sun Yat-sen University in Guangzhou, China. The paired non-tumor tissues were collected at least 2 cm away from the primary HCC. Both tumor and non-tumor tissues were histologically confirmed. All patients were unrelated ethnic Han Chinese who lived in Southeast China. None of the patients had received local or systemic anticancer treatments prior to the operation. This study was approved by the Institute Research Ethics Committee at the Third Affiliated Hospital of Sun Yat-sen University. Informed consent was obtained from each patient.

Cell lines derived from human hepatocellular carcinoma (MHCC97L, SMMC-7721, QGY-7703, BEL-7402, and BEL-7404) were obtained from the Cell Bank of the Chinese Academy of Sciences (Shanghai, China), and maintained in Dulbecco’s Modified Eagle’s Medium (DMEM, Life Technologies, Grand Island, NY, United States) supplemented with 10% fetal bovine serum (FBS, Hyclone, Thermo Fisher Scientific, Victoria, Australia).

### Immunohistochemistry (IHC)

Paraffin-embedded, formalin-fixed tissue was cut into 4-μm section. The samples were placed on polylysine-coated slide, de-paraffinized in xylene, rehydrated through graded ethanol, quenched for endogenous peroxidase activity in 0.3% hydrogen peroxide, and processed for antigen retrieval by microwave heating in 10 mM citrate buffer (pH 6.0). Sections were incubated at 4°C over-night with the polyclonal rabbit Ab against Fibulin-1, which was diluted 1:200 in Antibody Diluent with Background Reducing Components (DakoCytomation, Glostrup, Denmark). Immunostaining for Fibulin-1 was performed using ChemMate DAKO EnVision Detection Kit, Peroxidase/DAB, Rabbit/Mouse (code K 5007, DakoCytomation), which leaded to a brown-colored precipitate at the antigen site. Then, sections were counterstained with hematoxylin (Zymed Laboratories, South San Francisco, CA, United States) and mounted in non-aqueous mounting medium. All runs included a no primary antibody control.

All IHC staining was independently evaluated by two experienced pathologists in an effort to provide a consensus on staining patterns according to a scoring method. Fibulin-1 protein expression grading was conducted by analyzing the intensity and percentage of positive cells in five different areas. The intensity of positive signal was scored with a 4-point scale: 0 for no staining, 1 for mild staining, 2 for moderate staining, and 3 for intense staining. The percentage of positive cells was scored with a 4-point scale: 0 for no staining of cells in any microscopic areas, 1 for 1–30%, 2 for 30–60%, and 3 for over 60%. The total score of Fibulin-1 staining intensity was calculated by multiplying the intensity score by the percentage score. The relevant characteristics of the studied subjects are shown in Supplementary Table S1.

### Mice

Male BALB/c-nu mice were purchased from Vital River, Peking, China, and were bred in-house under specific pathogen-free (SPF) conditions. The animal experiments were performed according to the guidelines of the Animal Care and Use Committee of The Third Affiliated Hospital of Sun Yat-sen Universityand approved by the Animal Care and Use Committee of The Third Affiliated Hospital of Sun Yat-sen University.

### RNA Oligoribonucleotides and Plasmids

All RNA oligoribonucleotides were purchased from Genepharma (Shanghai, China). The siRNAs targeting the human *FBLN1* (GenBank accession no. NM_001996.3) and *NOTCH1* (NM_017617.4) mRNAs were denoted by FBLN1- siRNA1, FBLN1 siRNA2 and siNotch1, respectively. The NC RNA duplex for the siRNA was not homologous to any human genome sequences.

The coding sequence of the FBLN1 gene was PCR-amplified and inserted into the *Eco*RI/*Xba*I sites of the pcDNA3.1 vector (Life Technologies), as previously described (Su et al., 2009). All RNA oligoribonucleotides and the primers used for cloning are listed in Supplementary Table S2.

### Cell Transfection

RNA oligoribonucleotides were reverse transfected using Lipofectamine RNAiMAX (Invitrogen, Carlsbad, CA, United States) with a final concentration of 50 nM. The RNA transfection efficiency was approximately 70–80% using this method (Su et al., 2009; Gong et al., 2013). The cells were transfected with the plasmid DNA using Lipofectamine 2000 (Invitrogen).

### Apoptosis Analysis

Apoptosis was detected using a morphological examination and the TUNEL assay. As for the morphological examination, cells were stained with DAPI, and cells with condensed or fragmented nuclei were considered apoptotic cells. At least 750 cells in each sample were counted. TUNEL staining was performed using the *In Situ* Cell Death Detection Kit (Roche, Penzberg, Germany), according to the manufacturer’s protocol.

### RNA Isolation and Analysis of Gene Expression

RNA was isolated from 500 μl of serum using TRIzol LS Reagent (Life Technologies). The mRNA levels were evaluated by quantitative real-time PCR (qPCR) and protein levels were evaluated by western blotting. All serum samples were collected from patients at the Third Affiliated Hospital of Sun Yat-sen University.

### Extraction of FBLN1 Expression Levels and Clinical Dataset From TCGA and GEO

Gene expression profiling and clinicopathological data were obtained from TCGA HCC database as described previously (Jie et al., 2019). For survival analysis of FBLN1 expression in TCGA, UALCAN database draws a KM plot and show survival analysis results^1^. Expression data of GSE14520 in Gene Expression Omnibus (GEO^2^) was selected and obtained owing to the large HCC tissue samples and complete clinical information.

### Bioinformatics and Immune-Related Analysis

Fibulin-1-related gene sets were submitted to the LinkedOmics website^3^ to perform KEGG pathway analysis, based on TCGA database. The association between the Fibulin-1 and Notch1 was analyzed in TCGA HCC cohort using GEPIA^4^. We employed TIMER to estimate the proportion of immune cell types in a mixed cell population online^5^. An online tool—xCell^6^ —was used to analyze the fraction of stromal and immune cells in tumor samples. TISIDB was also adopted to explore the correlation between Fibulin-1 expression and abundance of immune infiltrates^7^.

### Statistical Analysis

Data were expressed as the means ± standard errors of the means (SEM) from at least three independent experiments. The student’s *t*-test and the Mann-Whitney *U*-test for analyses were used for analyzing the difference between two groups, and Kruskal–Wallis tests for more than two groups. Survival curves were obtained using the Kaplan-Meier method with the log-rank test. The analyses were performed using SPSS software (version 13.0, SPSS Inc., Chicago, IL, United States). A *p*-value < 0.05 was used as the criterion for statistical significance, and all statistical tests were two-sided.

## Results

### Fibulin-1 Is Frequently Overexpressed in HCC Tissues

**FIGURE 1:**
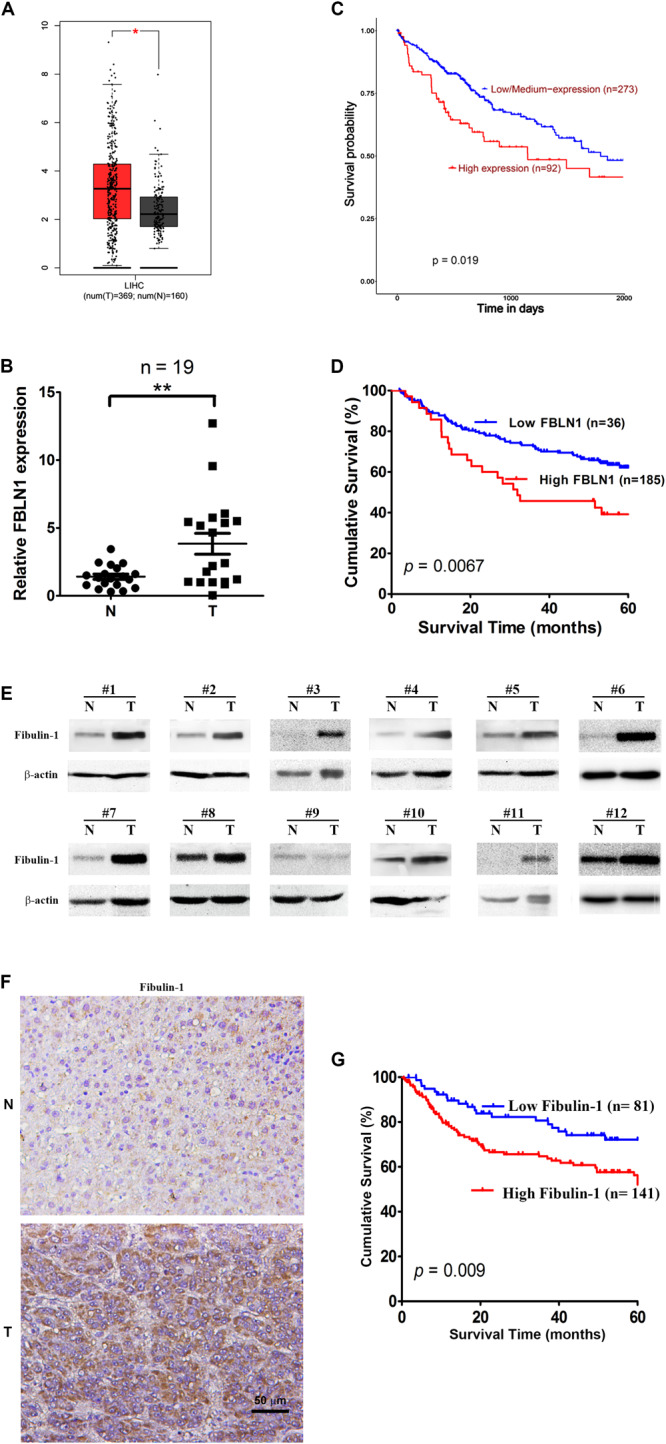
Elevated expression of both mRNA and protein levels of Fibulin-1 are associated with poor survival in HCC patients. **(A)** Comparison of Fibulin-1 expression between HCC cancer tissues and non-cancerous tissues involved in TCGA based on GEPIA. LIHC, hepatocarcinoma; *p < 0.05. **(B)** Fibulin-1 is significantly upregulated in HCC tissues. Fibulin-1 levels were analyzed in 19 paired HCC and adjacent non-tumor tissues using real-time qPCR. The Fibulin-1 level in each sample was normalized to the β-actin level. T, HCC tissue; N, adjacent non-tumor tissues. The median Fibulin-1 level in all examined samples was set to 1. **p < 0.01. **(C,D)** Influence of Fibulin-1 expression on the overall survival of patients with HCC expressing high Fibulin-1 levels and low Fibulin-1 levels, as analyzed using the Kaplan-Meier analysis with TCGA **(C)** or GEO **(D)** database, respectively. **(E)** Western blots of the Fibulin-1 protein in 12 paired HCC tissues (T) and the matched adjacent non-tumor tissues (N) from the same patients. **(F)** Representative immunohistochemical staining for Fibulin-1 in HCC tissue (down) and adjacent non-tumor tissue (up). Fibulin-1-positive cells displayed brown staining in the periphery and cytoplasm. The scale bar represents 50 μm. T, HCC tissue; N, adjacent non-tumor tissues. **(G)** Influence of Fibulin-1 expression on the overall survival of 141 patients with HCC expressing high Fibulin-1 levels and 81 patients with HCC expressing low Fibulin-1 levels, as evaluated using the Kaplan-Meier analysis.

Based on TCGA database, Fibulin-1 was highly overexpressed in HCC tissues compared with normal tissues (Figure 1A). To validate the results of TCGA analysis, a real-time PCR analysis was performed to analyze the expression levels of Fibulin-1 in 19 paired HCC and adjacent non-tumor liver tissues. Our findings showed that Fibulin-1 was upregulated in 84.2% (16/19) of HCC tissues compared with the adjacent non-cancerous tissues (Figure 1B). We hypothesized that the increased Fibulin-1 expression contributed to pathogenesis and predicted that patients with HCC expressing higher levels of Fibulin-1 would exhibit worse clinical outcomes. By using UALCAN based on TCGA data, we found that patient with tumors in the higher Fibulin-1 expression group had reduced survival^1^ (Figure 1C). This result was validated in GSE14520 dataset with more than 200 HCC samples. As for GSE14520, we stratified patients into low and high Fibulin-1 groups based on the expression data using the “minimum *P*-value” approach by X-tile software. Importantly, Fibulin-1 has significant relationship with patients survival rate (Figure 1D). The above results suggested that Fibulin-1 mRNA level may be a valuable prognostic factor in HCC. Moreover, multivariate analysis revealed that Fibulin-1 correlated with a significant 1.7-fold increased risk of death for HCC patients with the high Fibulin-1 level vs the low level (*p* = 0.001, Supplementary Table S3).

Moreover, the protein level of Fibulin-1 was also significantly increased in HCC tissues compared with that in adjacent non-tumor liver tissues, as shown in the western blot analysis (Figure 1E). Next, the important prognostic role of Fibulin-1 in HCC from TCGA and GEO database was further confirmed with our own samples. The characteristics of the studied individuals are illustrated in Supplementary Table S1. A total of 222 patients were stratified into two groups (low and high groups) based on the expression data obtained from our immunohistochemical staining experiments (Figure 1F). Consistent with above results, the overall survival rate was significantly lower in patients with high Fibulin-1 expression than in patients with low Fibulin-1 expression (Figure 1G). On univariate and multivariate analysis, the high Fibulin-1 expression showed significant higher probability of death (*p* = 0.001, Table 1). Thus, Fibulin-1 is frequently overexpressed at the levels of mRNA and protein in human HCC tissues and is an independent predictor for death.

**TABLE 1:** Univariate and Multivariate Analysis of Factors Associated with Overall Suvival with our collected tissues^a^.

**Clinical variable**	**Hazard ratio (95% CI^c^)**	***P*-value**
**Univariate analysis^b^**		
Fibulin-1 (High vs Low)	2.1(1.2−3.5)	**0.006**
Gender (M vs F)	0.8(0.5−1.6)	0.840
Age-yr (>40 vs ≤40)	1.4(0.6−3.2)	0.463
HBV (Positive vs Negative)	1.6(0.7−3.7)	0.269
Cirrhosis (Yes vs No)	2.1(1.0−4.2)	**0.041**
Ascites (Yes vs No)	1.5(0.7−3.2)	0.252
AFP (≥400 vs <400 ng/mL)	1.1(0.7−1.8)	0.565
ALT (≥50 vs <50 U/L)	0.8(0.5−1.4)	0.552
Tumor size (>5 cm vs ≤5 cm)	2.1(1.1−3.8)	**0.016**
Multinodular (Yes vs No)	1.4(1.1−1.9)	**0.014**
Tumor capsule (None/imcomplete vs Complete)	1.3(0.8−2.0)	0.356
Portal vein tumor thrombus (Yes vs No)	1.8(1.0−3.3)	**0.051**
**Multivariate analysis^d^**		
Fibulin-1 (High vs Low)	2.4(1.4−4.2)	**0.001**
Cirrhosis (Yes vs No)	2.4(1.2−4.9)	0.015
Tumor size (>5 cm vs ≤5 cm)	2.3(1.3−4.2)	0.007

*Bold indicates significant values. ^*a*^: analysis was performed on the entire cohort (*n* = 222). ^*b*^: Univariate analysis, Cox proportional hazards regression. ^*c*^: 95% CI, 95% confidence interval. ^*d*^: Multivariate analysis, Cox proportional hazards regression.*

### Analysis of the Function of Fibulin-1 in Apoptosis

**FIGURE 2:**
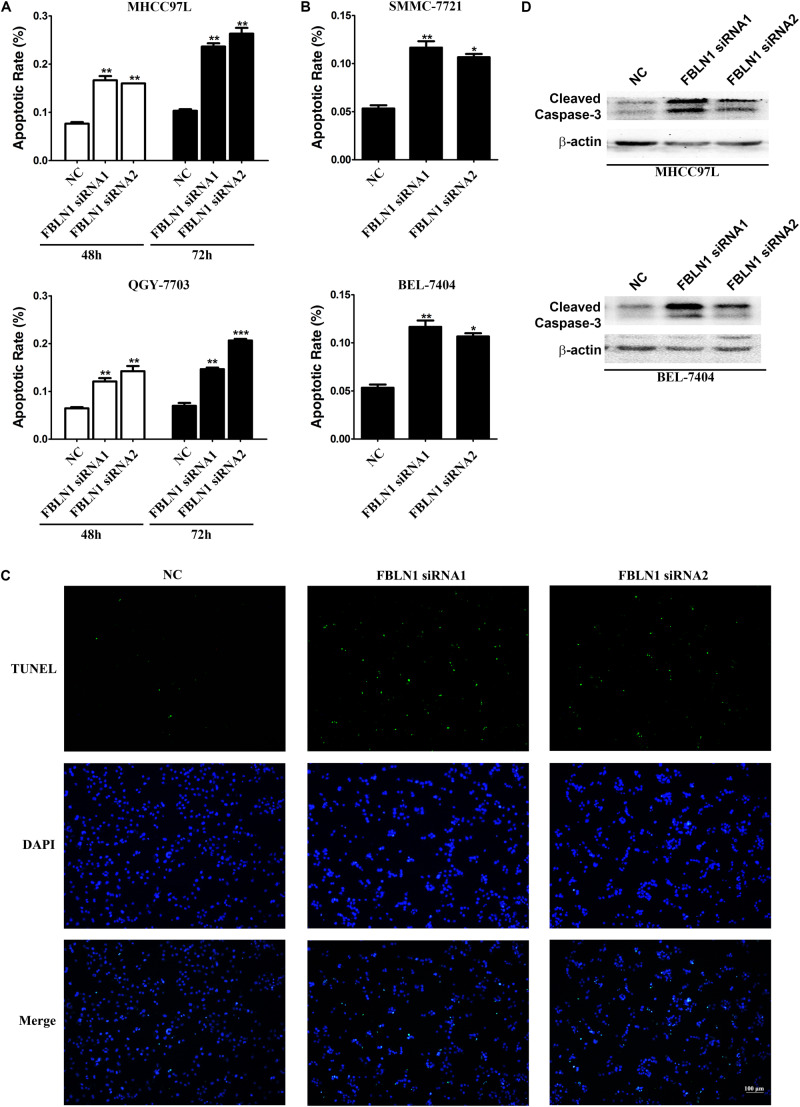
A reduction in endogenous Fibulin-1 expression promotes apoptosis in various cancer cell lines. **(A,B)** The inhibition of Fibulin-1 expression promotes apoptosis upon nutrient starvation. MHCC97L, QGY-7703, SMMC-7721, and BEL-7404 cells were transfected with FBLN1 siRNA1, FBLN1 siRNA2 or NC for the indicated times. Twenty-four hours after transfection, MHCC97L and QGY-7703 cells were deprived of serum for 48 h or 72 h **(A)**. SMMC-7721 and BEL-7404 cells were deprived of serum for 72 h **(B)**. Apoptosis was analyzed using DAPI staining. *p < 0.05; **p < 0.01; ***p < 0.001. **(C)** Evaluation of apoptosis using TUNEL staining. Twenty-four hours after transfection, MHCC97L cells were deprived of serum for 48 h and then subjected to TUNEL staining. Apoptotic cells displayed green staining. Scale bar, 100 μm. **(D)** The inhibition of Fibulin-1 expression increased the cleaved caspase-3 levels. Twenty-four hours after transfection, MHCC97L and BEL-7404 cells were deprived of serum for 48 h, followed by detection of the cleaved caspase-3 levels using western blotting. β-actin, internal control.

Rapidly growing tumors, such as HCC, often exhibit an insufficient blood supply. Thus, solid cancer cells should develop resistance to nutrient starvation-induced apoptosis. Therefore, we analyzed whether Fibulin-1 affected the apoptosis of cancer cells grown in nutrient-deprived medium. MHCC97L, SMMC-7721, QGY-7703, and BEL-7404 cell lines were transfected with a negative control (NC) or Fibulin-1 siRNA, which obviously decreased the level of endogenous Fibulin-1 (Supplementary Figure S1), to explore the effect of Fibulin-1 on apoptosis. According to the morphological examination, the downregulation of Fibulin-1 expression using RNAi significantly increased apoptosis in all four cell lines compared with that in the NC (Figures 2A,B). Next, the pro-apoptotic effect of Fibulin-1 silencing shown in the morphological analysis described above was further confirmed using terminal deoxynucleotidyl transferase-mediated nick-end labeling (TUNEL) staining (Figure 2C). Fibulin-1 silencing also induced the activation of caspase-3 (Figure 2D).

**FIGURE 3:**
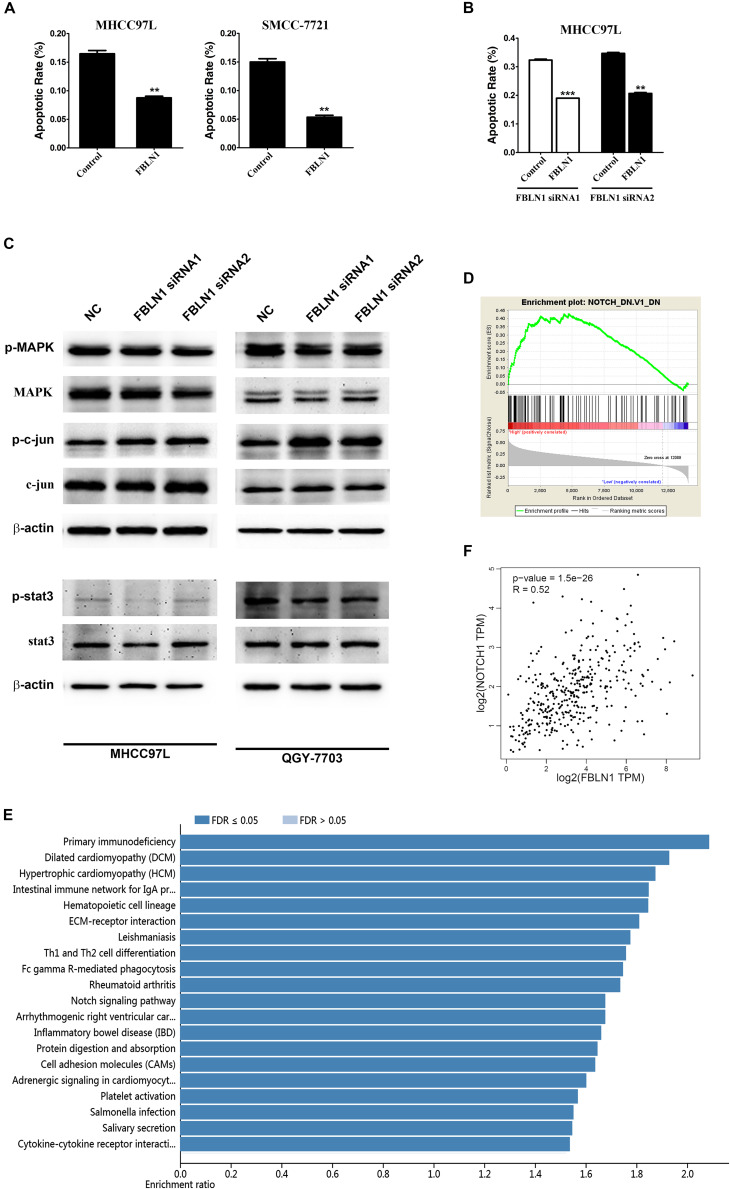
Fibulin-1 overexpression decreases apoptosis in various cancer cell lines and bioinformatic analysis of Fibulin-1. **(A)** Twenty-four hours after transfection with the control (pcDNA3.1) or FBLN1 plasmid (pcDNA3.1-FBLN1C), MHCC97L and SMCC-7721 cells were deprived of serum for 72 h. **(B)** Twenty-four hours after transfection with FBLN1 siRNA1 or FBLN1 siRNA2, MHCC97L cells were transfected with the control (pcDNA 3.1) or FBLN1 plasmid (pcDNA3.1-FBLN1C) for the next 24 h. Then, the MHCC97L cells were deprived of serum for 48 h. Apoptosis was analyzed using DAPI staining. **p < 0.01; ***p < 0.001. **(C)** A reduction in the endogenous Fibulin-1 levels did not affected the endogenous p-MAPK, p-c-jun and p-stat3 levels in MHCC97L and QGY-7703 cells. Twenty-four hours after transfection, MHCC97L and QGY-7703 cells were deprived of serum for 48 h, and then the levels of endogenous proteins were detected using western blotting. β-actin, internal control. **(D)** Gene set enrichment analysis (GSEA) of the genes relevant to Fibulin-1 in HCC based on TCGA database. GSEA for gene sets related with Fibulin-1 expression. **(E)** KEGG pathway enrichment of Fibulin-1. KEGG pathway analysis was applied to investigate the pathways that FBLN1-related genes mainly regulated. Pathways with a *p*-value < 0.05 was significant. KEGG pathway analyses was performed from TCGA database via LinkedOmics (http://www.linkedomics.org). **(F)** The association between the Fibulin-1 and Notch1 was analyzed in TCGA HCC cohort using GEPIA (http://gepia.cancer-pku.cn/index.html).

A gain-of-function analysis was performed in the MHCC97L and SMMC-7721 HCC cell lines to confirm the results from the loss-of-function analysis. First, we detected the expression of different Fibulin-1 transcripts in each tumor and non-tumor pair using a real-time PCR analysis, and then the tumor-to-non-tumor ratios of Fibulin-1 expression were calculated. The Fibulin-1C mRNA was the variant that exhibited the greatest upregulation in tumor tissues, followed by transcript D (Supplementary Figure S2), whereas transcripts A and B were undetectable (data not shown). MHCC97L cells transfected with pcDNA3.1-Fibulin-1C (FBLN1C) which encoded the entire coding sequence of Fibulin-1C but lacked the 3’-untranslated region (UTR), exhibited an obvious increase in Fibulin-1 expression (Supplementary Figure S3). In response to serum deprivation, Fibulin-1-overexpressing cells displayed significantly reduced apoptosis rates compared with the control cells (Figure 3A). Notably, Fibulin-1 overexpression abrogated apoptosis induced by Fibulin-1 silencing (Figure 3B).

Based on these results, Fibulin-1 silencing promotes apoptosis in our cell models, and Fibulin-1 overexpression may lead to cellular resistance to apoptosis induced by nutritional deficiency.

### Mechanisms by Which Fibulin-1 Silencing Induced Apoptosis

We next explored the molecular mechanisms by which siFibulin-1 promoted apoptosis. The p-MAPK, p-c-jun and p-stat3 showed no obvious changes after stimulation, when β-actin was used as a control (Figure 3C). Therefore, we utilized gene set enrichment analysis (GSEA) and Kyoto Encyclopedia of Genes and Genomes (KEGG) pathway analysis to analyze the pathways that Fibulin-1 mainly regulated with TCGA database. GSEA indicated that the gene set of NOTCH was enriched in HCC samples with Fibulin-1 highly expressed (Figure 3D). Consistent with this result, Fibulin-1 in the KEGG enrichment analysis was related to NOTCH signaling pathway (Figure 3E). Therefore, the association between the Fibulin-1 expression and Notch1 was analyzed. Fibulin-1 level had significant correlation with the Notch1 expression based on TCGA database with Gene Expression Profiling Interactive Analysis (GEPIA) (Figure 3F).

**FIGURE 4:**
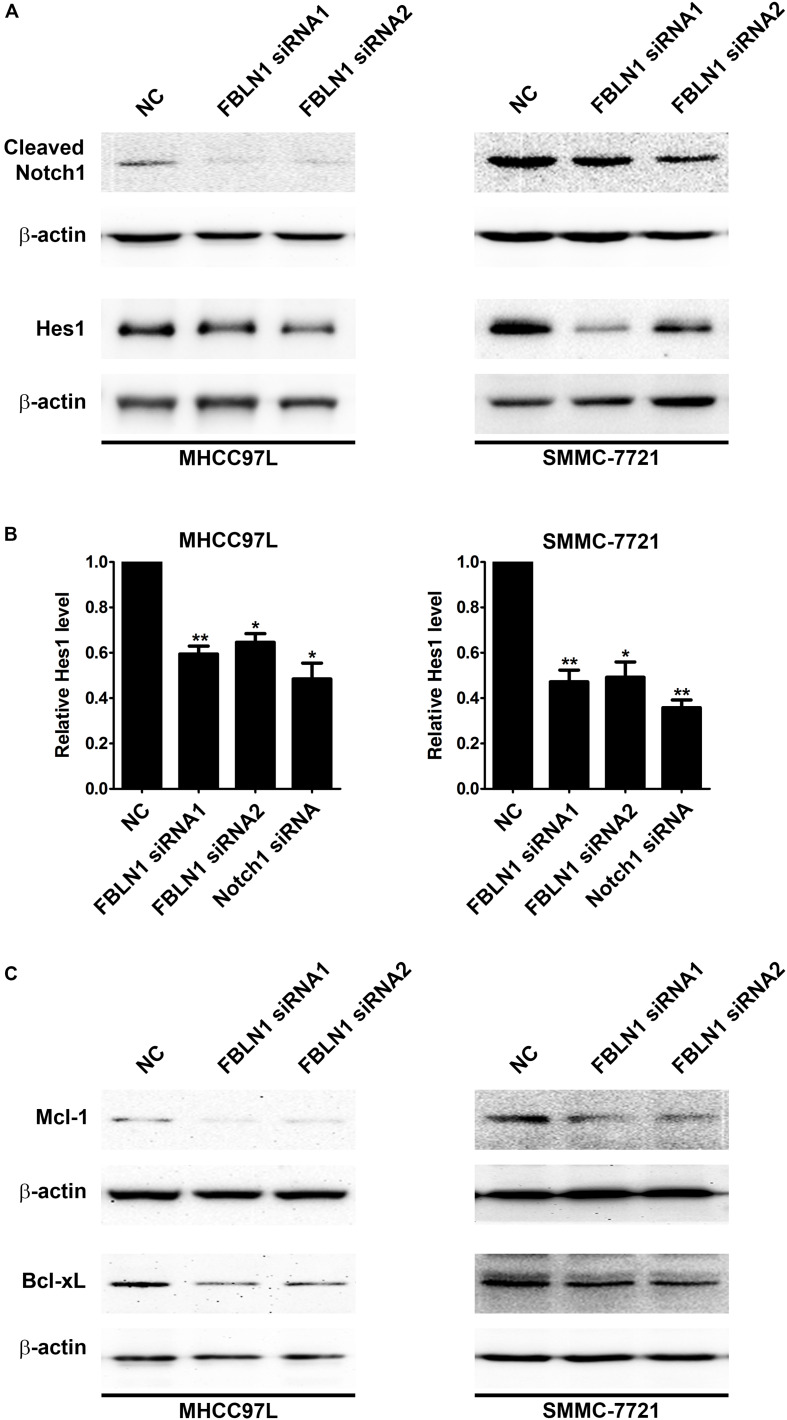
Inhibition of Fibulin-1 expression promotes apoptosis by regulating Notch signaling. **(A,C)** A reduction in the endogenous Fibulin-1 levels decreased the endogenous cleaved Notch1, Hes1, Mcl-1 and Bcl-xL levels in MHCC97L and SMMC-7721 cells. Twenty-four hours after transfection, MHCC97L and SMMC-7721 cells were deprived of serum for 48 h, and then the levels of endogenous proteins were detected using western blotting. β-actin, internal control. **(B)** The inhibition of Fibulin-1 expression decreased the level of the Hes1 mRNA. Seventy-two hours after transfection, MHCC97L and SMMC-7721 cells were subjected to real-time qPCR. The level of Fibulin-1 in each sample was normalized to the β-actin expression. No error bar is shown for NC transfectant because its normalized Fibulin-1 level was set to 1. *p < 0.05; **p < 0.01.

**FIGURE 5:**
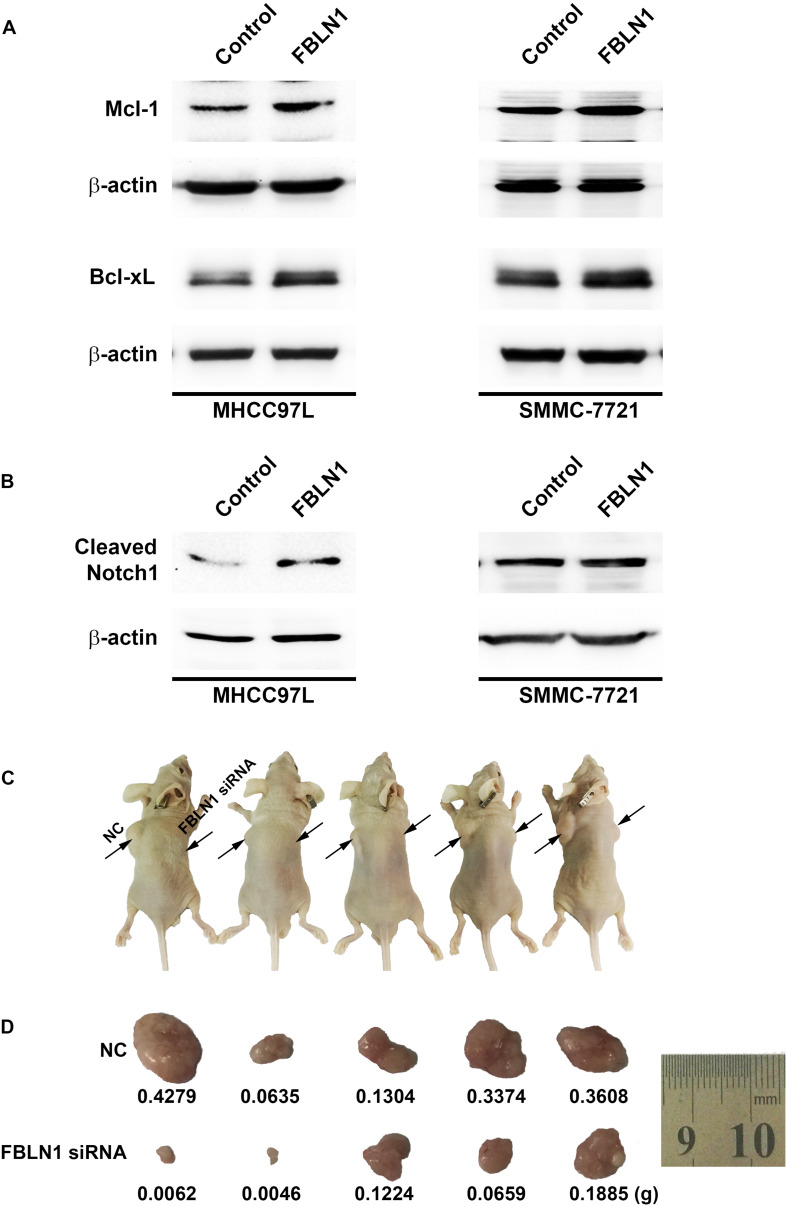
The impact of Fibulin-1 silencing on tumorigenecity. **(A,B)** Fibulin-1 overexpression increased the Mcl-1, Bcl-xL and cleaved Notch1 levels. Twenty-four hours after transfection, MHCC97L and SMMC-7721 cells were deprived of serum for 48 h, and cellular proteins were analyzed by western blotting. β-actin, internal control. **(C,D)** Tumor formation in nude mice. Representative photographs **(C)** and the tumor weights **(D)** are shown. The notations NC and Fibulin-1 silencing shown in **C** indicate the flanks injected with NC-transfected and Fibulin-1 siRNA-transfected cells, respectively.

Furthermore, the domains of Fibulin-1 were analyzed to determine the mechanism. It has been reported that Fibulin-3 activates Notch signaling and promotes tumor growth in a Notch-dependent manner (Hu et al., 2012). Because Fibulin-1 and Fibulin-3 share conservation of the Delta-Serrate-Lag motif, a characteristic of the extracellular domain of Notch ligands (Supplementary Figure S4), we speculated that Fibulin-1 might promote Notch1 cleavage and increase the levels of the active Notch-1 intracellular domain (NICD). Subsequently, the effect of siFibulin-1 on the endogenous expression of its potential target genes was examined. Fibulin-1 knockdown decreased Notch-1 cleavage and obviously decreased the NICD level (Figure 4A), whereas Fibulin-1 overexpression increased the NICD levels in MHCC97L cells and leaded to a slightly increase in SMMC-7721 cells (Figure 5B). Furthermore, siRNA-mediated knockdown of Fibulin-1 downregulated the Notch-dependent expression of the Hes1 protein and mRNA (Figures 4A,B).

Notch not only sustains the Mcl-1 expression level but also increases the Bcl-xL level and, in turn, promotes cell survival in multiple cancers (Wang et al., 2006, 2010; Fassl et al., 2012). Mcl-1 and Bcl-xL, anti-apoptotic members of the Bcl-2 family, are frequently upregulated in various tumor types (Takehara et al., 2001; Sieghart et al., 2006). Interestingly, Fibulin-1 knockdown obviously attenuated Mcl-1 and Bcl-xL proteins levels in MHCC97L and SMMC-7721 cells (Figure 4C), while Fibulin-1 overexpression consistently upregulated Mcl-1, Bcl-xL and NICD levels (Figures 5A,B). To explore the role of Notch1, Mcl-1 and Bcl-xL in Fibulin-1-inhibited apoptosis, we analyzed whether silencing of these genes could antagonize the anti-apoptotic effects of Fibulin-1. siRNA duplexes targeting Notch1, Mcl-1 or Bcl-xL attenuated the expression of their respective target genes (Supplementary Figure S5A). pcDNA3.1-Fibulin-1C was co-transfected with siRNA duplexes targeting Notch1, Mcl-1 and Bcl-xL, followed by serum starvation for 72h. Compared with NC-transfected cells, siNotch1, siMcl-1 and siBcl-xL transfectants displayed higher levels of apoptosis (Supplementary Figure S5B). An *in vivo* model was used to further confirm these findings. NC- and Fibulin-1 siRNA-transfected BEL-7402 cells were subcutaneously (s.c.) injected into either scapula of the same nude mice, respectively. The mice were followed and xenograft growth was observed for 5 weeks. The silencing of Fibulin-1 in BEL-7402 cells significantly reduced the tumor volume (Figures 5C,D and Supplementary Figure S6). Thus, Fibulin-1 significantly increases tumorigenicity *in vivo* and *in vitro*.

**FIGURE 6:**
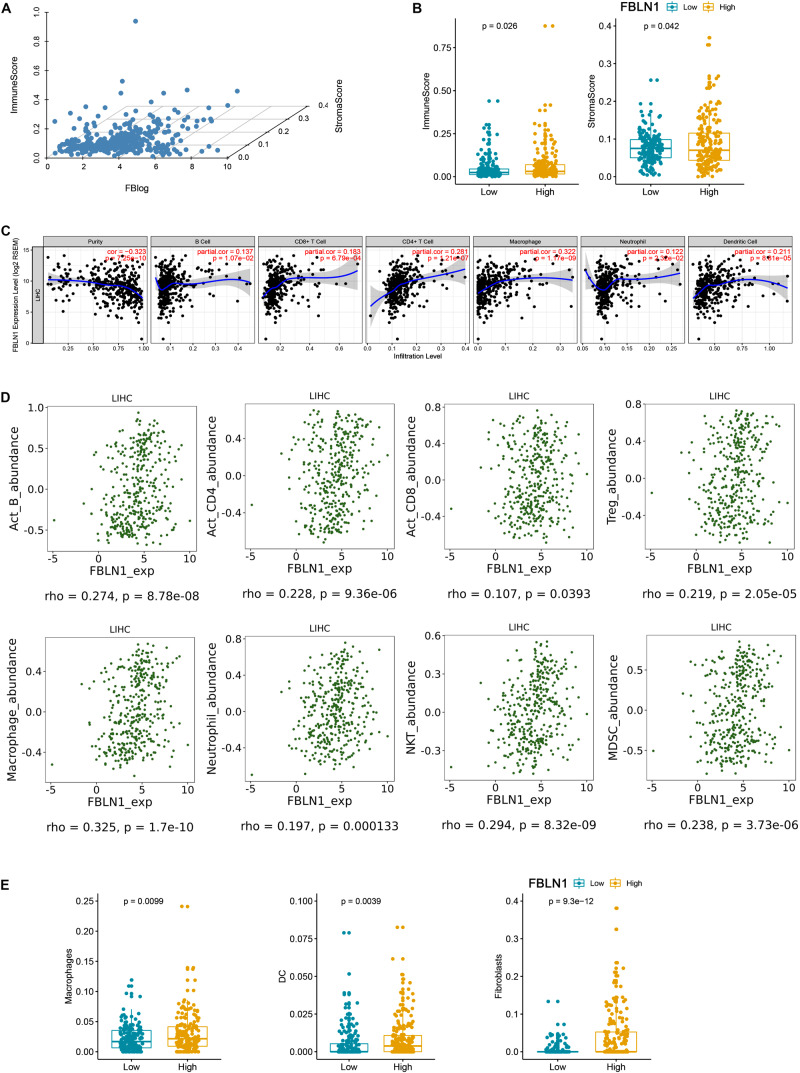
There is a positive correlation between Fibulin-1 and immune cell and extracellular matrix cell infiltrates. **(A)** xCell immune score, xCell stromal score and Fibulin-1 gene expression were showed based on TCGA HCC data. **(B,E)** HCC subjects in TCGA database were divided into two groups based on the median value of Fibulin-1, and the group with high Fibulin-1 showed significantly increased **(B)** xCell immune score and xCell stromal score or **(E)** macrophage, dendritic cells and fibroblasts. **(C)** Immune cell landscape of HCC compared with TCGA gene expression of Fibulin-1. Scatter plots were drew using the online tool TIMER to identify the correlation between Fiblin-1 and different profiles of immune cells. Each dot represents a single tumor sample. **(D)** Relations between abundance of tumor-infiltrating immune cell types and FBLN1 were identified using online tool TISIDB.

Furthermore, we analyzed the correlation between Fibulin-1 and immune cell and extracellular matrix cell infiltrates using xCell. xCell could estimate the abundance scores of immune cell types and extracellular matrix cells (Aran et al., 2017). According to the TCGA database, Fibulin-1 expression, immune score and stromal score were showed in Figure 6A. Furthermore, we divided HCC patients into low and high Fibulin-1 groups based on median value of FBLN1 expression, and found that the immune and stromal score was higher in the high group than in the low group (Figure 6B). Furthermore, it is remarkable that the elevation of tumor purity (i.e., the percentage of cancer cells in a solid tumor sample) was negatively correlated with the expression of FBLN1 by using the TIMER web server (Figure 6C). Specifically, this may be due to an increase in the numbers of infiltrating macrophages, B cells, CD4 + cells, dendritic cells and neutrophils, and FBLN1 expression was associated with increased infiltrating macrophages, CD4 + cells and dendritic cells (Figure 6C). To confirm the results of TIMER web server, we used the online tool TISIDB and found the positive correlation between Fibulin-1 expression and infiltrating macrophages (Figure 6D). Furthermore, the high macrophage, dendritic cells and fibroblasts was observed in the high Fibulin-1 group than in the low Fibulin-1 group by xCell (Figure 6E).

### The Expression Levels of Circulating Fibulin-1 RNA Were Upregulated in the Serum of HCC Patients

**FIGURE 7:**
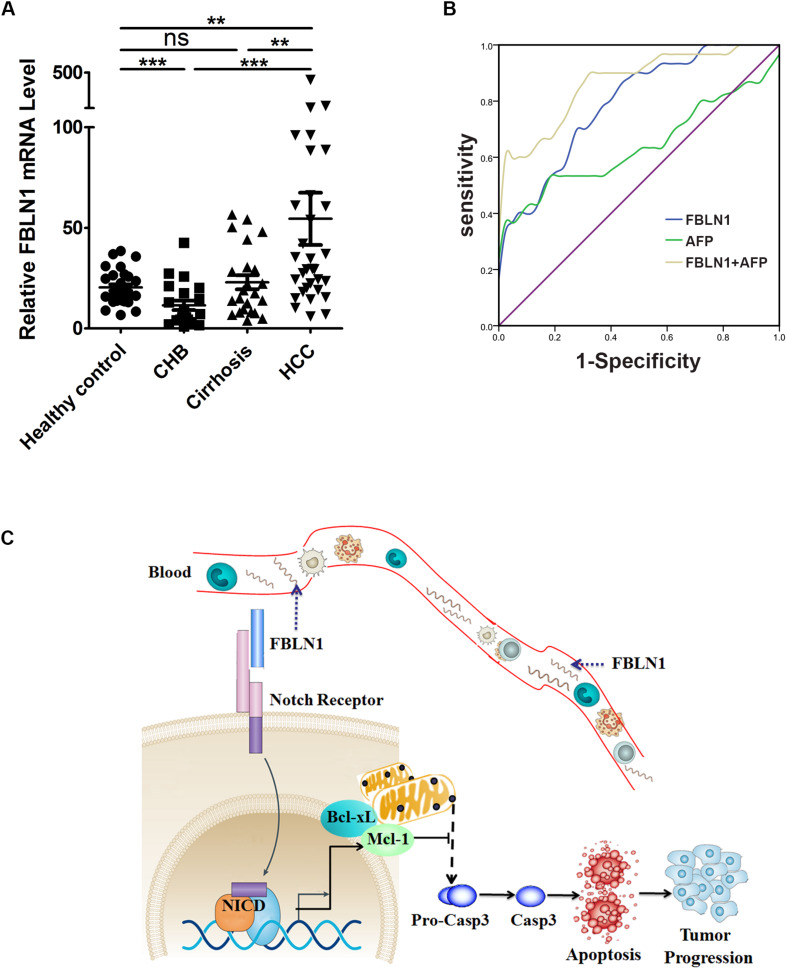
The serum levels of Fibulin-1 messenger RNA were significantly higher in patients with HCC. **(A)** Serum Fibulin-1 levels in the patient groups and healthy controls. FBLN1 mRNA levels were measured in different subgroups of patients and healthy controls. HC: healthy control, CHB: chronic hepatitis B, HCC: hepatocellular carcinoma. **p < 0.01; ***p < 0.001. **(B)** ROC curves for FBLN1 and AFP for all patients with HBV-related HCC compared with those for patients with HBV-related cirrhosis or chronic liver disease. **(C)** Schematic showing the regulatory network of FBLN1 and its target genes in apoptosis.

Since Fibulin-1 plays an important role in apoptosis, and increased expression of the Fibulin-1 gene indicates a worse prognosis in patients with HCC, we further investigated whether Fibulin-1 was a diagnostic biomarker for HCC. Serum samples were collected from four groups of participants: healthy controls (HC, *n* = 30), patients with chronic hepatitis B (*n* = 21), patients with hepatitis B virus (HBV)-related cirrhosis (*n* = 23), and patients with HBV-related HCC (*n* = 31) (Supplementary Tables S4, S5). The ages of the healthy control and HCC groups were well matched (*P* = 0.2739). The serum levels of Fibulin-1 messenger RNA were significantly higher in patients with HCC than in the control groups (mean 54.51 ± 73.47 vs 20.38 ± 8.333). Importantly, the Fibulin-1 levels were also increased in patients with HCC (54.51 ± 73.47) compared with that in patients with cirrhosis (22.93 ± 16.87) and that in patients with chronic hepatitis B (11.53 ± 10.76) (Figure 7A). We next evaluated the performance of Fibulin-1 compared with α-fetoprotein (AFP) in discriminating patients with HCC from at-risk controls. Fibulin-1 had a greater area under the receiver operating characteristic curve (AUROC) (0.791; 95% confidence interval (CI): 0.690–0.893) than AFP (0.640; 95% CI: 0.498–0.782), suggesting that Fibulin-1 is superior to AFP at diagnosing HCC (Figure 7B). At a cut-off of 20 IU/ml, the sensitivity of AFP was 0.6 and the specificity was 0.512. The optimal diagnostic cut-off value of Fibulin-1 based on the ROC curve was 22.38, with a sensitivity of 0.7 and a specificity of 0.744. Furthermore, we examined whether a combination of these biomarkers would improve the diagnostic performance for patients with HCC. When this combined score was compared with AFP alone in diagnosing HCC, the AUROC was improved (0.868 vs 0.640), suggesting that the combination of these biomarkers significantly improves the diagnosis of HCC compared with either test alone. The role of serum Fibulin-1 levels in patients with AFP-negative (<20 IU/ml) HCC was also investigated. Approximately 35.48% (11/31) of patients with HCC had normal AFP levels. Among the patients with AFP-negative HCC, 90.91% (10/11) exhibited elevated Fibulin-1 levels using the optimal diagnostic cut-off of 22.38.

Based on our results, a significant upregulation of Fibulin-1 expression at both mRNA and protein levels was a frequent event and was associated with shorter overall survival. Furthermore, a higher Fibulin-1 level in HCC tissue decreased apoptosis by downregulating Mcl-1 and Bcl-xL expression (Figure 7C). More importantly, Fibulin-1 may be a serum diagnostic marker for patients with HCC.

## Discussion

Although abnormal Fibulin-1 expression is frequently observed in different types of cancer, the biological function of dysregulated Fiublin-1 expression is largely cell context-dependent. The role of Fibulin-1 in regulating apoptosis currently remains unclear. In this study, we frequently observed an upregulation of Fibulin-1 expression in HCC tissues and a negative correlation between an increased Fibulin-1 level and a poor prognosis. Moreover, Fibulin-1 is an independent predictor for death. Fibulin-1 silencing significantly increased apoptosis in liver cancer cell lines upon nutrient starvation by downregulating Mcl-1 and Bcl-xL expression. Fibulin-1 is a multi-faceted molecule that may promote or inhibit apoptosis, depending on the cellular context. The opposite effects mediated by Fibulin-1 in different cell types may provide a basis for targeted therapy if the underlying mechanisms are disclosed.

Epithelial cells must be attached to the ECM to survive. However, during tumorigenesis and cancer development, cancerous epithelial cells must evolve a variety of strategies to circumvent apoptosis in the absence of the ECM. Given the harsh tumor microenvironment, such as nutrient deprivation and hypoxia, tumor cells must employ multiple mechanisms to evade apoptosis, escape clearance by anticancer therapy or immune surveillance systems. These mechanisms include the functional loss of the p53, increased anti-apoptotic molecules (Mcl-1 and Bcl-xL), decreased pro-apoptotic molecules and short-circuiting of the extrinsic ligand-induced death pathway. Mcl-1 and Bcl-xL could bind to pro-apoptotic molecules such as Bax and Bak1, preserve the integrity of the mitochondria, and thus decrease cell death. Mcl-1 and Bcl-xL are frequently increased in various tumor types and promote tumor progression as pro-survival molecules (Takehara et al., 2001; Sieghart et al., 2006). Fibulin-1 silencing suppressed Mcl-1 and Bcl-xL expression by targeting Notch signaling. Fibulin-1 knockdown induced caspase-3-dependent apoptosis. Thus, Mcl-1 and Bcl-xL are the predominant mediators of Fibulin-1 siRNA-induced apoptosis in liver cancer cells, although other unidentified targets may also be involved.

The mechanism by which Fibulin-1 exerts both apoptosis-promoting and inhibiting functions is still unclear. Fibulin-1 is downregulated by promoter hypermethylation and promotes apoptosis in gastric cancer and hepatitis C virus (HCV)-associated HCC(Kanda et al., 2011). However, Fibulin-1 suppresses doxorubicin-induced apoptosis in breast cancer, implying that the biological role of Fibulin-1 is largely cell context-dependent. As shown in the present study, Fibulin-1 silencing in the different cancer cell lines promoted apoptosis. Furthermore, Fibulin-1 expression is highly and specifically upregulated in HCC patients and correlates with reduced patient survival. Thus, tumor cells may upregulate Fibulin-1 expression to survive in the harsh environment, emphasizing the potential of Fibulin-1 as a relevant target in the tumor microenvironment.

Notch signaling is conserved throughout the animal kingdom (Bray, 2006). The Notch pathway plays a crucial role in diverse developmental and physiological processes. Importantly, Notch activity also emerges as a contributing factor to many cancer types, including HCC. Although Notch signaling has been suggested to inhibit HCC, Notch overexpression and activation have been shown to promote tumor progression in the liver and promote the formation of liver tumors in mice (Villanueva et al., 2012; Kunnimalaiyaan et al., 2015). The NICD is cleaved and released upon binding of a Notch ligand to the Notch extracellular domain. The NICD then enters the cell nucleus to modify gene expression, including the transcription factor Hes1 (Bray, 2006). Hes1 plays an important role in the Notch signaling pathway. Notch has been reported to sustain Mcl-1 expression, promote cell survival in multiple cancers, and increase the Bcl-xL level (Wang et al., 2006, 2010). Here, Fibulin-1 silencing suppressed Hes1 expression in HCC cell lines and its targets levels, Mcl-1 and Bcl-xL. Furthermore, Fibulin-1 overexpression increased the Mcl-1 and Bcl-xL levels.

Curative operations are often more successful in patients with HCC that is detected at an early stage. A simple blood test for early detection of HCC has been the dream of all researchers and physicians. Circulating biomarkers in the serum may provide more insights into this possibility. In our research, the serum Fibulin-1 levels were significantly increased in HCC patients than in healthy controls, chronic hepatitis B patients and individuals with HBV-induced liver cirrhosis. The serum Fibulin-1 levels were significantly more sensitive at distinguishing HCC patients from at-risk controls than the AFP levels. Based on our findings, Fibulin-1 is a promising biomarker for HCC surveillance.

In conclusion, we investigated the biologic role of Fibulin-1 in apoptosis and the underlying mechanisms. Upregulation of Fibulin-1 expression may confer malignant transformation and promote tumor progression, suggesting a potential application for Fibulin-1 silencing in anticancer therapy.

## Data Availability Statement

All datasets generated for this study are included in the article/Supplementary Material.

## Ethics Statement

The animal study was reviewed and approved by Institute Research Ethics Committee at the Third Affiliated Hospital of Sun Yat-sen University.

## Author Contributions

YC and BH conceived and designed the work. JG, YJ, and CX performed research, collected and analyzed the data. XL and YC collected human tissue samples. YW, JC, QZ, and ZG provided technical assistance. JG and YJ wrote the manuscript. All authors read and approved the final manuscript.

## Conflict of Interest

The authors declare that the research was conducted in the absence of any commercial or financial relationships that could be construed as a potential conflict of interest.

## Footnotes

1 http://ualcan.path.uab.edu/cgi-bin/ualcan-res.pl

2 http://www.ncbi.nlm.nih.gov/geo/

3 http://www.linkedomics.org

4 http://gepia.cancer-pku.cn/index.html

5 https://cistrome.shinyapps.io/timer/

6 http://xcell.ucsf.edu/

7 http://cis.hku.hk/TISIDB/

## Abbreviations

ECM: extracellular matrix
FBLN1: Fibulin-1
HCC: hepatocellular carcinoma
NICD: Notch-1 intracellular domain.
