# Supplementary material for: Increased Expression of Fibulin-1 Is Associated With Hepatocellular Carcinoma Progression by Regulating the Notch Signaling Pathway
Source: Front Cell Dev Biol. 2020 Jun 16;8:478. doi: 10.3389/fcell.2020.00478 (PMC7308487; doi:10.3389/fcell.2020.00478)
Supplement: Supplementary file 1 [file Data_Sheet_1.docx]

**Supplementary Tables**

**Table S1.** Clinicopathological parameters of 222 patients with HCC

|  | | |
| --- | --- | --- |
| **Parameters** | **N(%) or Mean(SD)** |  |
| Man | 192(86.4%) |  |
| Age(years) | 48.0±11.9 |  |
| HBsAg positive | 222(100%) |  |
| Cirrhosis | 175（78.8%） |  |
| AFP≤20 ng/ml | 64（28.8%） |  |
| AFP≤400 ng/ml | 121（54.5%） |  |
| Cell differentiation |  |  |
| Low | 19(8.6%) |  |
| Moderate | 186(83.8%) |  |
| High | 17(7.6%) |  |
| Survival time |  |  |
| <5 years | 151(68.0%) |  |
| ≥5 years | 71(32.0%) |  |

**Table S2.** Sequences of RNA and DNA Oligonucleotides

| **Name** | **Sense Strand/Sense Primer (5' - 3')** | **Antisense Strand/Antisense Primer (5' - 3')** |
| --- | --- | --- |
| **MiRNA and siRNA Duplexes** | | |
| FBLN1 siRNA1 | GACAAUAGCUGCAAAGAUAdTdT | UAUCUUUGCAGCUAUUGUCdCdT |
| FBLN1 siRNA2 | CUAGGCAACUGUAUUGAUAdTdT | UAUCAAUACAGUUGCCUAGdAdG |
| Notch1 siRNA | CACCAGUUUGAAUGGUCAAdTdT | UUGACCAUUCAAACUGGUGdGdA |
| NC | UUCUCCGAACGUGUCACGUdTdT | ACGUGACACGUUCGGAGAAdTdT |
|  |  |  |
| **Primers for Cloning (Restriction enzyme sites were underlined)** | | |
| FBLN1 | AGTGAATTCGCCACCATGGAGCGCGCCGCGCCGT | AGTTCTAGATCAGAGCTCTGCAGACACAAAGA |
|  |  |  |
| **Primers for RT-PCR** | |  |
| FBLN1 | GGCCACTCATCAGAAGGACT | ACAGTGCAGCTCCTCCAG |
| FBLN1C | CCGCTGCCTGGCCTTCGA | CCTCCTCATTGCCGCCG |
| FBLN1D | CCGCTGCCTGGCCTTCGA | CCGCAGGTTCCCTTCCG |
| FBLN1A | TCCATCAACGAGACCTGCTT | GTTGGTCATGAACAGCAGCA |
| FBLN1B | TCCATCAACGAGACCTGCTT | GCTATGCTTCATCTGCTGGG |
| Hes1 | AGAAGGCGGACATTCTGGAA | ACCTCGGTATTAACGCCCTC |
| β-actin | GCACCCAGCACAATGAAGAT | ACATCTGCTGGAAGGTGGAC |

| **Table S3.** Univariate and Multivariate Analysis of Factors Associated with Overall Suvival with GSE14520 database^a^ | | |
| --- | --- | --- |
| Clinical Variable | Hazard Ratio  (95% CI ^c^) | *p*  Value |
| **Univariate analysis ^b^** |  |  |
| FBLN1 (High vs Low) | 2.0 (1.2-3.2) | **0.008** |
| Gender (M vs F) | 1.7 (0.8-3.5) | 0.154 |
| Age(>45 vs ≤45) | 1.1 (0.7-1.7) | 0.770 |
| ALT(≥50 vs <50 U/L) | 1.1 (0.7-1.7) | 0.726 |
| TNM(II/III vs I ) | 3.0(1.8-4.9) | **<0.001** |
| BCLC (B + C vs 0+A) | 3.5(2.3-5.5) | **<0.001** |
| AFP (≥300 vs <300 ng/mL) | 1.6 (1.1-2.4) | **0.042** |
| Cirrhosis (Yes vs No) | 4.6(1.1-18.8) | **0.032** |
|  |  |  |
| **Multivariate analysis ^d^** |  |  |
| FBLN1 (High vs Low) | 1.7 (1.1-2.9) | **0.031** |
| TNM (II/III vs I) | 2.0 (1.1-3.4) | **0.015** |
| Cirrhosis (Yes vs No) | 4.1 (1.0-16.8) | **0.048** |
| BCLC (B + C vs 0+A) | 2.5 (1.5-4.1) | **<0.001** |
| Note: Bold indicates significant values. | | |
| ^a^: analysis was performed on the entire cohort (n=222). | | |
| ^b^: Univariate analysis, Cox proportional hazards regression. | | |
| ^c^: 95% CI, 95% confidence interval. | | |
| ^d^: Multivariate analysis, Cox proportional hazards regression | | |

**Table S4.** Eligibility criteria for the recruited participants

| **Group** | **Criteria** |
| --- | --- |
| Healthy control | 1. Absence of HBV markers (HBsAg, HBeAg, anti-HBe, and anti-HBc) |
|  | 2. No liver disease and other systematic disease |
|  | 3. Persistently normal AST and ALT levels |
| Chronic hepatitis B† | 1. HBsAg-positive>6 months |
|  | 2. Serum HBV DNA>10^5^ copies/mL |
|  | 3. Persistent or intermittent elevation in AST or ALT levels |
| Liver cirrhosis‡ | 1. With chronic hepatitis B |
|  | 2. Liver biopsy indicates pseudolobuli, portal hypertension, or in the stage IV |
|  | 3. If no biopsy available, diagnosis must be supported by two imaging technologies |
| Hepatocellular carcinoma | 1. Diagnosed based on at least two imaging technologies (hepatic ultrasound together with CT and/or MRI) |
|  | 2. Confirmed histopathologically by two independent pathologists § |
|  | 3. No pre-operative chemotherapy, radiotherapy, transarterial chemoembolization or ablation before collection of blood samples |

HBV, hepatitis B virus; HBsAg, HBV surface antigen; HBeAg, HBV e antigen; anti-HBe, antibody against HBeAg.; anti-HBc, HBV core antibody; AST, aspartate aminotransferase; ALT, alanine aminotransferase. †From the American Association for the Study of Liver Diseases (AASLD) Practice Guidelines (2009) (Lok AS, Hepatology 2009, 50: 661-62). ‡According to the description in Lancet (Schuppan D, Lancet 2008, 371: 838-51), and the stage of liver biopsy was according to the Metavir system. §All the hepatocellular carcinoma cases were further confirmed histopathologically according to AASLD guidelines (Bruix J, Hepatology 2005, 42: 1208-36).

**Table S5.** Clinical characteristics of studied subjects

|  | | **Healthy controls (n=30)** | | **CHB patients (n=21)** | | **Cirrhosis patients (n=23)** | | **HCC patients (n=31)** | |  |
| --- | --- | --- | --- | --- | --- | --- | --- | --- | --- | --- |
| Age(years) | 51.44±9.186 | | | 38.27±8.956 | | 47.09±11.19 | | 54.10±8.696 | |  |
| Gender[n(%)] |  | | |  | |  | |  | |  |
| Female | 1 | | | 3 | | 2 | | 5 | |  |
| Male | 29 | | | 18 | | 21 | | 26 | |  |
| AST(U/L) | 20.83±4.186 | | | 145.0±167.1 | | 105.6±66.85 | | 118.0±125.9 | |  |
| ALT(U/L) | 19.73±6.405 | | | 160.2±196.3 | | 73.78±53.83 | | 65.19±66.92 | |  |
| AFP | 6.4±1.62 | | | 108.8±150.2 | | 66.00±200.7 | | 463.7±545.1 | |  |
|  | | |  | |  | |  | |  | |

**Fig. S1**


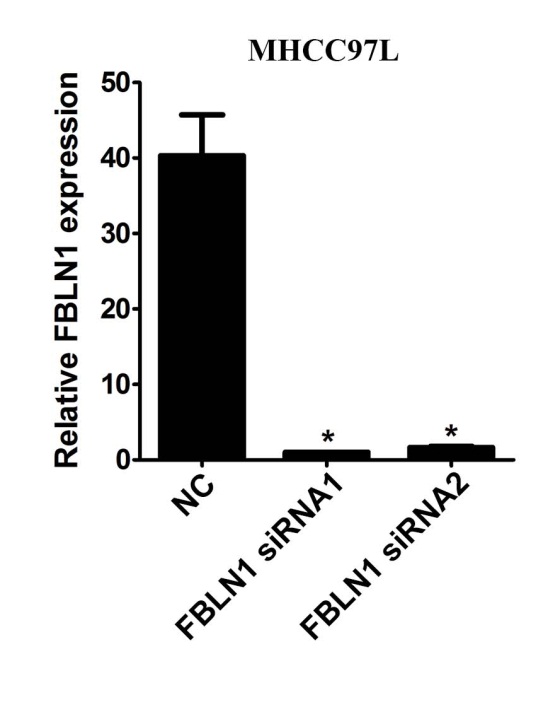


**Fig. S1.** Fibulin-1 siRNAs reduce the endogenous Fibulin-1 level. Seventy-two hours after transfection, MHCC97L cells were subjected to real-time qPCR. The Fibulin-1 level in each sample was normalized to the β-actin level. **p* < 0.05.

**Fig. S2**


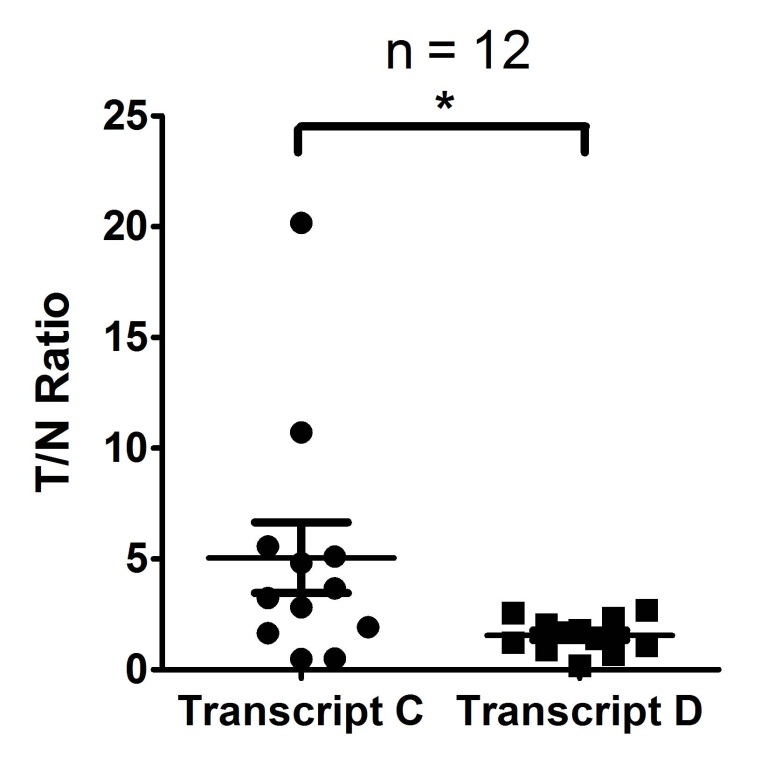


**Fig. S2.** The Fibulin-1C mRNA was the variant that was upregulated to the greatest extent in tumor tissues. The expression of different Fibulin-1 transcripts in each tumor and nontumor pair was detected using real-time qPCR analysis, and then the tumor-to-nontumor ratios of Fibulin-1 were calculated. The Fibulin-1 level in each sample was normalized to the β-actin level. **p* < 0.05.

**Fig. S3**


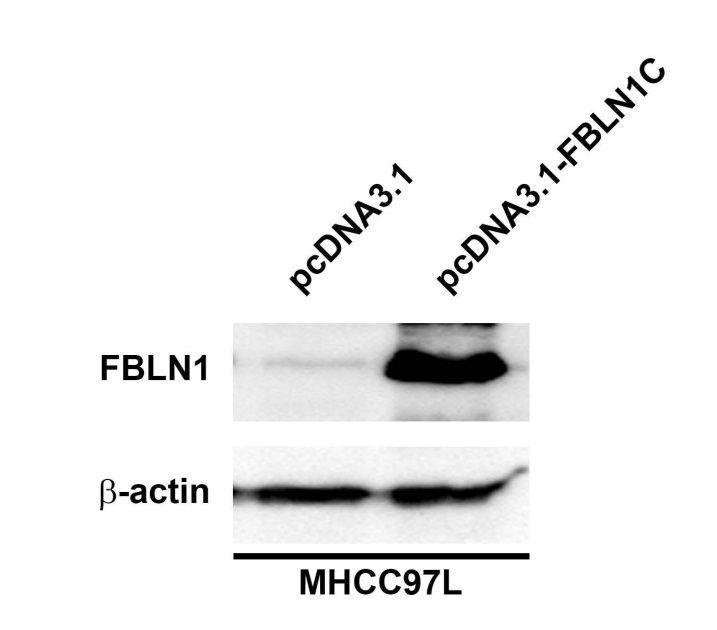


**Fig. S3.** pcDNA3.1-Fibulin-1C increased the Fibulin-1 level. Forty-eight hours after transfection, MHCC97L cells were analyzed by western blotting. β-actin, internal control.

**Fig. S4**


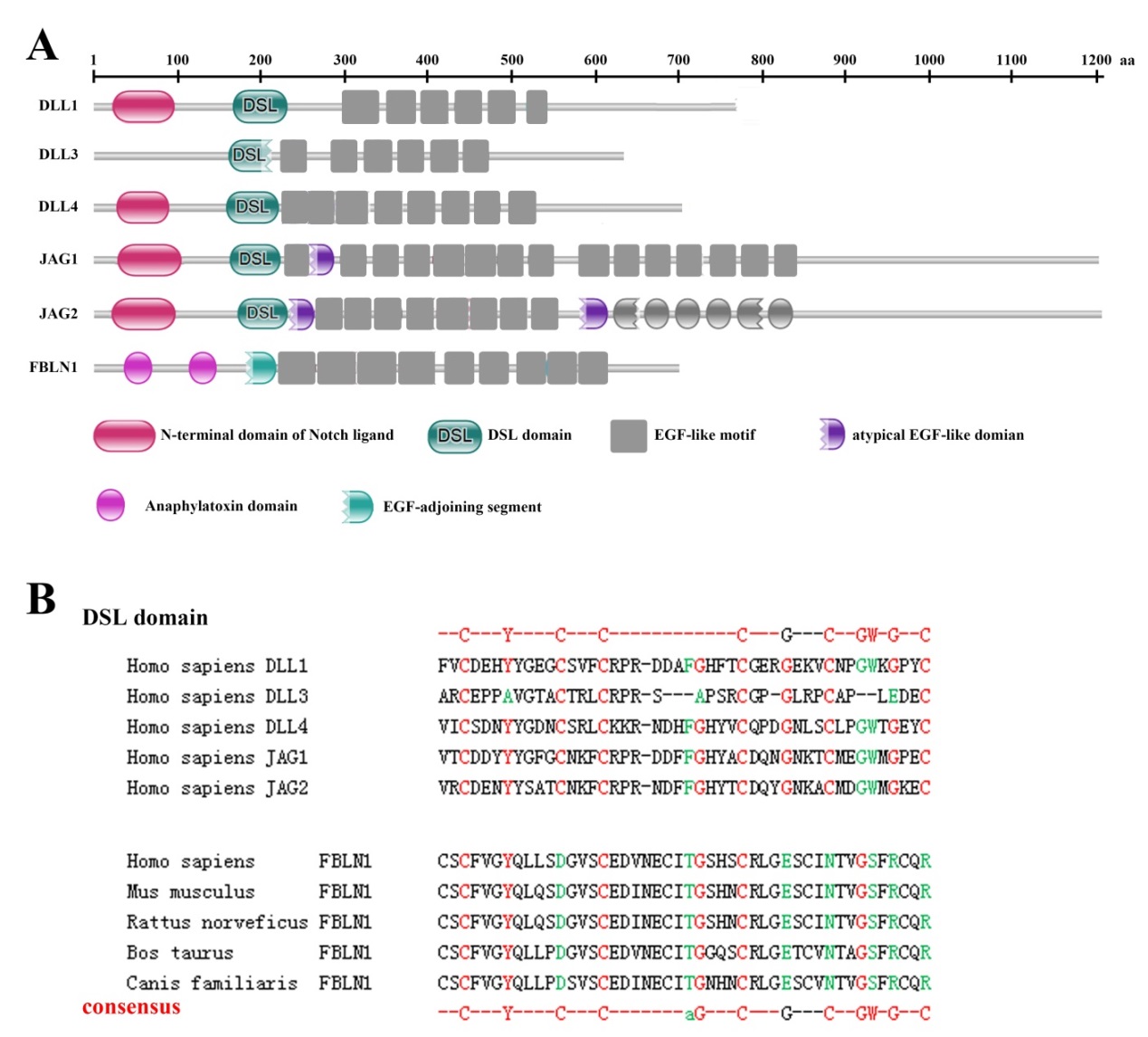


**Fig. S4.** (A) Conservation of the DSL motif in Fibulin-1 and canonical Notch ligands. Representation and alignment of canonical Notch ligands of the Delta and Jagged families together with Fibulin-1. B) Alignment (using ClustalW software) of the N-terminal sequences containing the Delta-Serrate-Lag (DSL) motif involved in Notch activation. Fibulin-1 contains 6 of the 11 aminoacids that form the DSL consensus. This sequence is highly conserved in fibulin-1 across species. a: acidic aminoacid. Aminoacids in red indicate conservation of the DSL residues between fibulin-1 and the Notch ligands while those in green show divergence from the consensus in fibulin-1 or in the Notch ligands themselves. aa: aminoacid.

**Fig. S5**


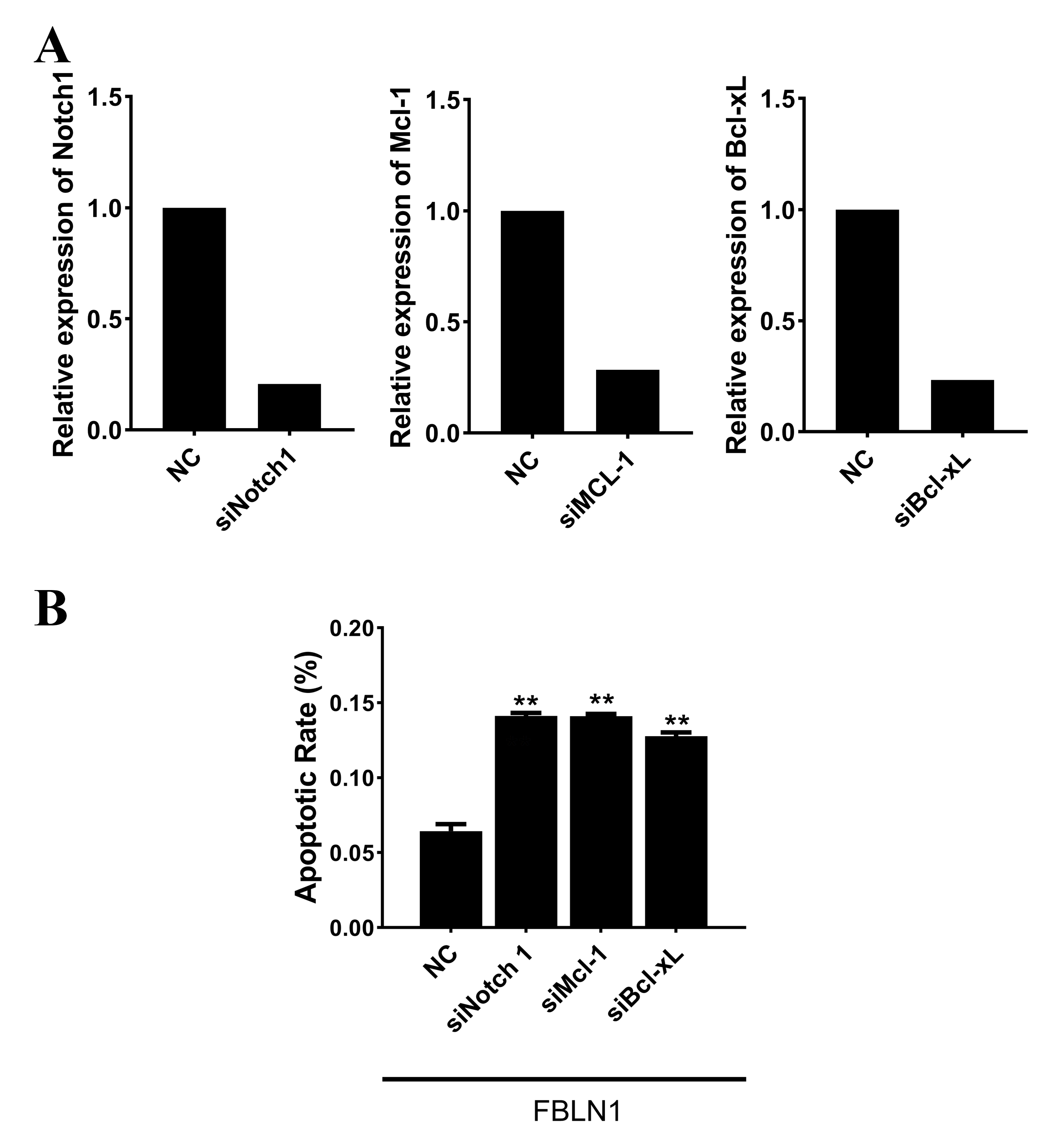


**Fig. S5.** (A) siNotch1, siMcl-1 and siBcl-xL reduce the endogenous each protein level. Seventy-two hours after transfection, SMMC-7721 cells were subjected to real-time qPCR. The mRNA level in each sample was normalized to the β-actin level. (B) pcDNA3.1-Fibulin-1C was co-transfected with siRNA duplexes targeting Notch1, Mcl-1 and Bcl-xL in SMMC-7721 cells, followed by serum starvation for 72 h. Apoptosis was analyzed using DAPI staining. ***p* < 0.01.

**Fig. S6**


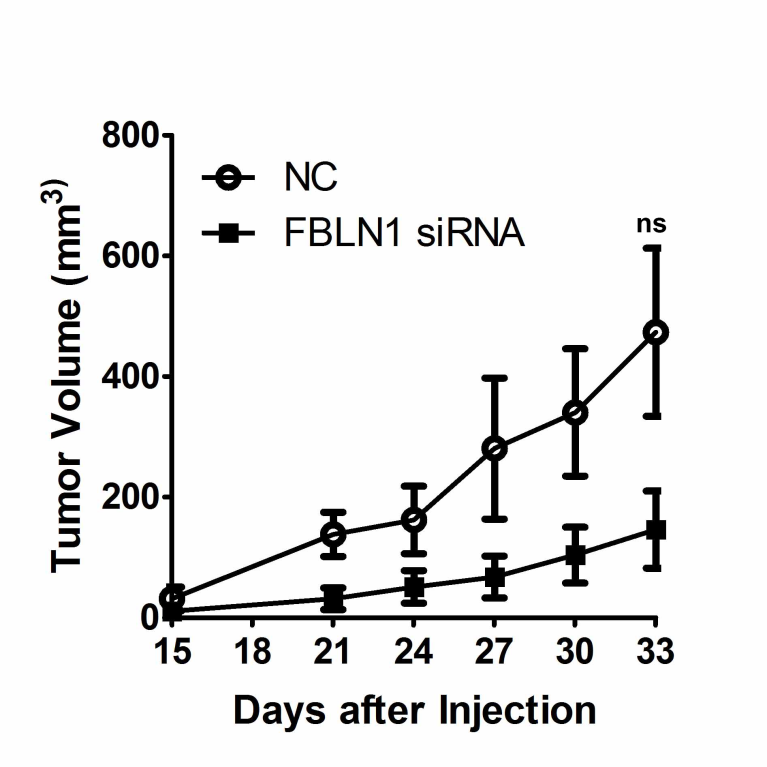


**Fig. S6.** The tumor growth curves are shown. NC and Fibulin-1 silencing indicate the flanks injected with NC-transfected and Fibulin-1 siRNA-transfected cells, respectively. ns, no significance.
